# Treatment of periprosthetic hip infection with retention of a well-fixed stem: six to 13-year outcomes

**DOI:** 10.1186/s42836-019-0002-8

**Published:** 2019-08-01

**Authors:** Takuya Otani, Hideki Fujii, Yasuhiko Kawaguchi, Tetsuo Hayama, Toshiomi Abe, Motoi Takahashi, Keishi Marumo

**Affiliations:** 1 Department of Orthopaedic Surgery, The Jikei University School of Medicine, 3-25-8 Nishi-Shinbashi Minato-ku, Tokyo, 105-8461 Japan; 2 Department of Orthopaedic Surgery, The Jikei University DAISAN Hospital, 4-11-1 Izumi-Honcho, Komae-shi, Tokyo, 201-8601 Japan

**Keywords:** Total hip arthroplasty (THA), Periprosthetic joint infection (PJI), Chronic infection, Treatment, Two-stage treatment, Antibiotic-loaded acrylic cement (ALAC), Implant retention

## Abstract

**Background:**

Treatment of periprosthetic joint infection (PJI) is challenging, generally requiring complete implant removal. However, recently reported treatments involve partial retention of implants because of the severe local and systemic burden on the patients and difficulties in functional preservation. Long-term results should be evaluated because of the risk of residual biofilm on the retained implant and late infection recurrence. We evaluated 6 to 13-year clinical outcomes of two-stage treatment of chronic PJI retaining well-fixed cementless stems.

**Methods:**

Among 36 surgeries for deep infection following hip arthroplasty performed from 2004 to 2011, six hips had a well-fixed and well-functioning cementless stem. These six hips were all chronic PJI and were treated without stem removal. The first-stage surgery involved acetabular cup removal and reconstruction by filling the acetabular defect with antibiotic-loaded acrylic cement, creating a socket-like hemispherical dent, and reducing the retained femoral head to this dent. After confirming infection eradication the second-stage acetabular reconstruction was performed. One patient died of an unrelated noninfective cause 1 year after the operation. Clinical outcomes of the remaining five patients were followed for 6 to 13 years.

**Results:**

Between the two surgeries (range; 2–5 months), patients underwent active range-of-motion and ambulation exercises. No dislocation was found during the interval. No recurrence of infection was found and good functional outcomes and radiographic findings were observed during the average follow-up of 109 months in all five patients.

**Conclusions:**

Two-stage treatment with retention of a well-fixed stem may minimize local and systemic burden of the patient and enhance functional preservation while obtaining long-term infection control. Although further study could establish the effectiveness and indications for this treatment option, currently used indications should be carefully evaluated considering factors including local and systemic conditions of the patient, implant fixation status, and type of bacteria.

## Background

The treatment of periprosthetic joint infection (PJI) is challenging and controversial. Various treatment options exist, but a two-stage treatment that involves the complete removal of implants in the first surgery and infection-free reconstruction in the second surgery is considered the gold standard for chronic PJI. The rationale for the complete implant removal is the difficulty in eradicating bacteria present inside the biofilm formed on the implants. According to previous studies, two-stage treatment eradicates infection in 79–96% of patients with PJI [1–5]. However, recent studies have investigated alternative treatment options mainly because of local and systemic burden on patients associated with the two-stage treatment, difficulty in preserving joint function during the interval between the two procedures, and temporal and financial burden of undergoing two surgeries. A classic treatment option is the one-stage treatment that comprises complete implant removal, thorough debridement, and joint reconstruction simultaneously. One-stage treatment was reported to eradicate infection in 56–100% of patients [6–10]. Problems associated with one-stage treatment are that the indications are currently unclear, infection eradication rate could be slightly lower compared with two-stage treatment, and invasiveness of the surgical procedure is extremely high.

Recently, several studies have reported a treatment strategy involving partial retention of implants as an additional treatment option for PJI: two-stage treatment with retention of well-fixed cementless implants [11–15], two-stage treatment with retention of well-fixed cement mantles [16], and single-stage treatment with retention of well-fixed implants [17]. These procedures were developed to preserve the function of the hip joint while reducing the local and systemic burden during treatment, but their detailed methods and effectiveness have not been verified or established. Because these types of specialized treatment are difficult to perform in a large-scale study, it is necessary to perform a relatively small-scale study (2–19 patients in the previous studies [11–17]) and accumulate treatment results. Additionally and more importantly, even though short-term treatment outcomes are preferable, these treatment options require long-term follow-up because these methods have the risk of residual biofilm and late infection recurrence.

Since 2004, we have performed a unique two-stage treatment for chronic PJI in patients with a well-fixed and well-functioning porous coated cementless stem on the femoral side. In this study, we describe the treatment procedure and postoperative outcome of patients whom we could follow-up for more than 6 years.

## Materials and methods

Between 2004 and 2011, 36 surgeries (involving 28 patients) were performed for deep infection following either bipolar hip arthroplasty (BHA) or total hip arthroplasty (THA) in our hospital. Of these patients, 6 had a well-fixed cementless stem with bone ingrowth into the porous coating [18], without any signs of loosening or malalignment of the stem on plain radiography and computed tomography. Moreover, no clinical complications, such as pain and dislocation, were observed, suggesting that these stems were well-functioning. All 6 patients were diagnosed with chronic infection [19], and the period between the onset of infection and surgery ranged from 2 to 20 (mean, 7) months. The diagnostic criteria for PJI published in 2013 [20] were used to retrospectively evaluate the diagnosis of the 6 patients. One of the patients had a chronic sinus tract that communicated with the joint, and methicillin-resistant *Staphylococcus aureus* (MRSA) was detected in multiple cultures. Another patient developed a subcutaneous abscess communicating with the joint, and the same bacterial species (*Staph. epidermidis*) was also detected after repeated culture. Two patients had a single positive culture (*Staph. capitis*), and positive histological, and serum biochemical findings. In two other patients, histological, serum biochemistry, and synovial fluid (white blood cell count) findings, but not culture findings, were positive. Sonication technique was not used in any of the tissue sampling. Histological findings were considered positive when ≥5 polymorphonuclear cells were observed in ≥5 high power fields [21]. Surgical procedures performed in all 6 patients during the first-stage surgery included removal of the acetabular cup, thorough intra-articular debridement involving partial or total resection of synovial layer from entire capsule, debridement of the bone-implant interface at the proximal end of the femoral stem macroscopically confirming clean bone ingrowth over the entire circumference, formation of a socket-like articulating spacer with a hemispherical dent by filling the acetabular defect with antibiotic-loaded acrylic cement (ALAC), and reducing the modular femoral head that had been sterilized intraoperatively and reassembled onto the neck of the stem. When creating a socket-like hemispherical dent as an articulating cement spacer, the height, lateral offset, and lateral and anterior opening angles were carefully set while checking the soft tissue tension. In patients with infection after BHA, the outer head was removed from the implant, and an articulating cement spacer was created in the same way after acetabular reaming. After infection control was confirmed by the negative CRP in the serum blood test and good clinical course the second-stage surgery was performed, which consisted of removal of the cement spacer, thorough debridement again, and subsequent acetabular reconstruction using antibiotics-loaded cement in all 6 cases and allograft and reinforcement device in 5 cases. All first- and second-stage surgeries were performed by the same surgeon. If the causative microorganisms were identified by culture, the cement spacer was mixed with antibiotics based on sensitivity test. In the cases with negative cultures, vancomycin (VCM) combined with broad-spectrum antibiotics against gram-negative bacteria was used. With respect to pre- and postoperative antibiotics protocols, adequate antibiotics were given for 3–5 days preoperatively in two patients who had already had multiple positive cultures. Other patients had no preoperative antibiotics in order to increase the chance to obtain positive intraoperative culture. After surgery, intravenous antibiotics in combination with oral rifampin were continued for 6 weeks. After second-stage reconstruction, intravenous antibiotics were continued for 1–2 weeks until blood examination (CRP) became negative with good clinical course. Subsequent oral antibiotics were not given in this series although it was added to the protocol in more recent years. Postoperative course was initially favorable in all 6 patients, but the oldest patient (82 years old; *Staph. capitis* infection after BHA) died of a cause unrelated to the infection 1 year after second-stage surgery. Up until her death, the patient was walking with a T-cane and showed no signs of recurrence of infection. The mean age of the remaining 5 patients (4 women, 1 man) was 64 (52–78) years at the time of first-stage surgery. These patients were relatively healthy and had no serious systemic complications. One of the patients had diabetes that was well-controlled with oral medication. Infection occurred after BHA (*n* = 1), THA (n = 1), THA after pelvic osteotomy (n = 1), and revision THA (*n* = 2). The types of cementless stems used in these patients were fully-porous coated cylindrical stem (*n* = 3, including 1 long stem) and proximally porous coated stem (n = 2). Mean follow-up duration after second-stage surgery was 109 (72–158) months. We evaluated the interval between the two surgeries, ambulatory function during the interval, recurrence of infection after reconstruction, and clinical and radiographic findings at the final examination. Functional outcomes were evaluated against the Japanese Orthopaedic Association scoring system (JOA score) [22] which evaluates hip joint function on a 100-point scale and consists of 40 points for pain, 20 for range of motion (ROM), 20 for ability to walk, and 20 for activities of daily living (ADL). Range of hip motion was recorded for flexion, abduction, internal rotation and external rotation. Loosening of implants and local osteolysis were radiographically assessed.

This study was conducted in accordance with the World Medical Association Declaration of Helsinki and was approved by the institutional review board of our institution. Written informed consent was obtained from each patient.

## Results

The mean interval between the two surgeries was 3.2 (2-5) months, during which patients participated in active range-of-motion and ambulation exercises. All the patients could walk with two crutches (touch-down weight bearing to 1/3 partial weight bearing). No dislocation or spacer displacement was observed during the interval. Furthermore, no recurrence of infection was observed during the 72–158 (mean, 109) month follow-up period after second-stage reconstruction. At the final examination, the mean total JOA score was 78 (35 for pain, 16 for range of motion, 13 for ambulation, and 14 for ADL). Range of hip motion evaluation revealed 89° for flexion, 28° for abduction, 18° for internal rotation, and 27° for external rotation. Radiographs at the final follow-up showed no signs of loosening or osteolysis.

### Case presentation

**Fig. 1:**
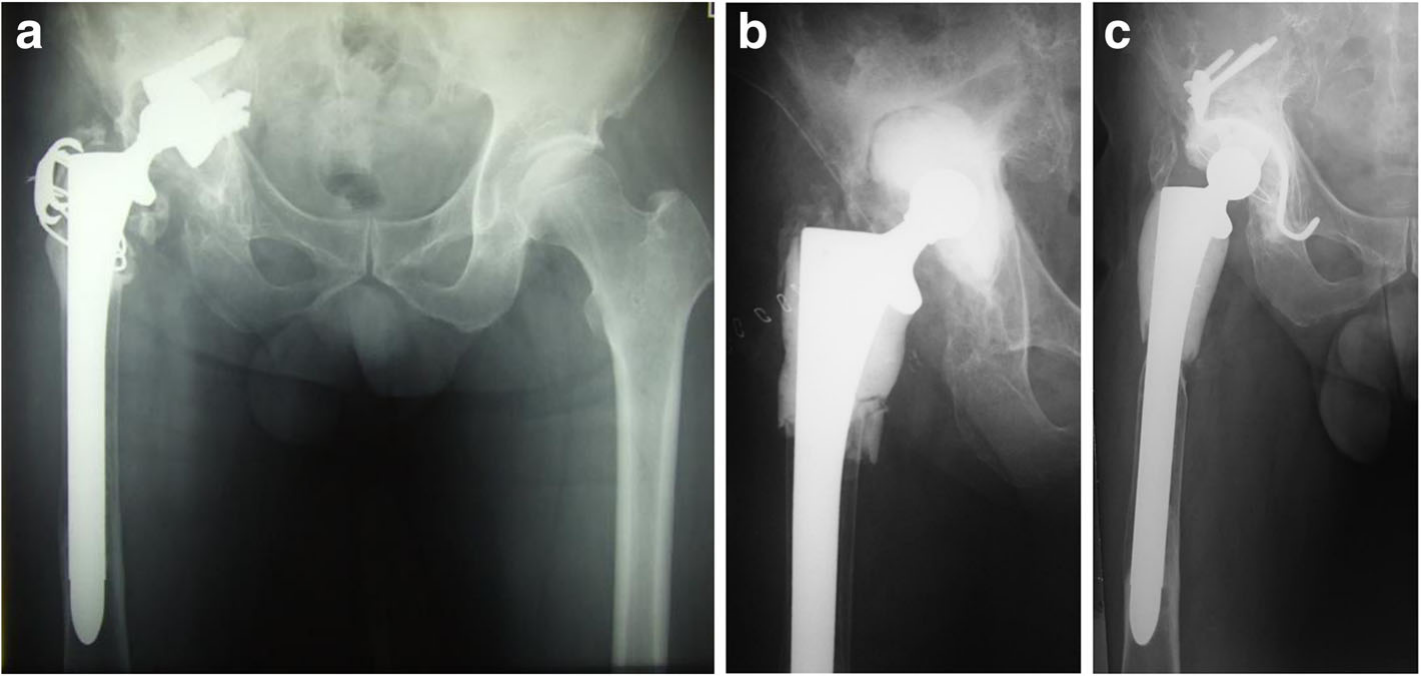
**a** A 65-year-old man developed late infection 3 years after revision THA. **b** The fully-porous coated long stem was retained and all the infected proximal femur was resected. Articulating cement spacer was made in the acetabular defect. **c** No signs of recurrence of infection were observed 158 months after the second-stage reconstruction

Case 1: A 65-year-old man who developed late infection 3 years after revision THA. Synovial fluid aspirate and intraoperative culture were both positive for *Staph. Epidermidis* (Fig. 1a). The fully-porous coated long stem was retained and we resected all the infected proximal femur (Fig. 1b). No signs of recurrence of infection were observed during a period of 158 months after the second-stage reconstruction (Fig. 1c).

**Fig. 2:**
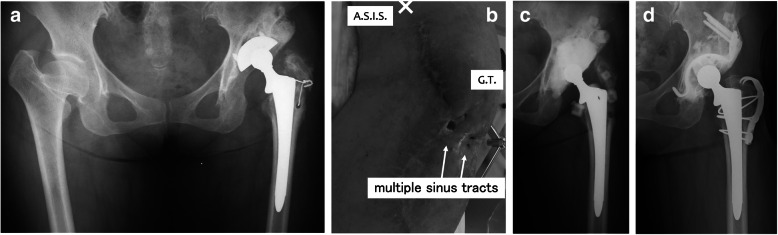
**a** A 52-year-old woman developed deep MRSA infection after THA. **b** The patient was referred to our department after four repeated irrigation and debridement procedures having developed multiple chronic sinus tracts in her thigh. **c** Hydroxyapatite spacers in addition to articulating cement spacer were used to contain antibiotics in this patient. **d** No signs of recurrence of infection were observed 94 months after the second-stage reconstruction

Case 2: A 52-year-old woman developed deep MRSA infection after THA that was performed 4 years after pelvic osteotomy (Fig. 2a). Despite four repeated irrigation and debridement procedures, the previous surgeon failed to achieve suppression of infection and the patient was referred to our department having developed multiple chronic sinus tracts in her thigh (Fig. 2b). In this patient, 12 hydroxyapatite spacers (containing 1 g of VCM) (BONECERAM-P; Olympus Terumo Biomaterials Co., Tokyo) were used in addition to ALAC (containing 2 g of VCM) (Fig. 2c). The tissue cultures at the second-stage surgery were all negative. Ninety-four months after the second-stage reconstruction, the patient had a good postoperative course with no signs of recurrence of infection (Fig. 2d).

## Discussion

Reports of various stem removal techniques abound, including extended trochanteric osteotomy [24], Gigli saw, trephine reamer, and the window technique [25], yet the removal of well-fixed cementless stems remains extremely difficult with inevitable significant damage to the proximal femur. Thus, it is difficult to apply an endoprosthesis-like articulating cement spacer to the destroyed proximal femur and preserve hip function until the second-stage reconstruction. Furthermore, recent studies have shown that postoperative mortality rate among patients who underwent implant removal surgery is as high as 24–45% [4, 5, 23], indicating that this type of surgery exerts heavy systemic burden on patients, in addition to local damage to the femur. In the treatment of infection of all types, including PJI, it is always important to maintain healthy local as well as systemic conditions, hemodynamically and immunologically. Minimizing surgical invasiveness is thus, an important challenge that surgeons need to address in the treatment of PJI.

**Fig. 3:**
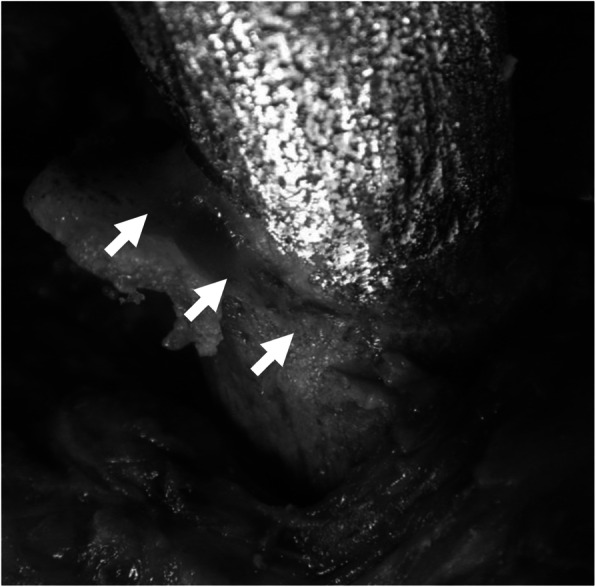
Intraoperative findings of the Case 1; Thorough debridement of the proximal femur was performed using a high-speed burr until fresh, clean bone ingrowth was identified circumferentially

Another rationale for retaining well-fixed stems is that bone ingrowth is thought to function as a barrier to microbial invasion. Today, almost all cementless stems are circumferentially-porous coated over the proximal portion. This makes the removal of well-fixed stems difficult; however, circumferential bone ingrowth may function as a barrier against intramedullary invasion of bacteria. The surgeon should carefully examine the entire circumference of the bone-implant interface and perform thorough debridement using tools such as a high-speed burr until fresh, clean bone ingrowth can be identified (Fig. 3). Possibility of stem retention could be increased when this aggressive and circumferential debridement of bone-implant interface is combined with a thorough debridement of the entire synovial capsule. Successful retention of a well-fixed stem could reduce both local and systemic burden on patients, enabling preservation of hip function between the two-stage surgeries, which could contribute to the accomplishment of ultimate goal of long-term clinical success.

**Fig. 4:**
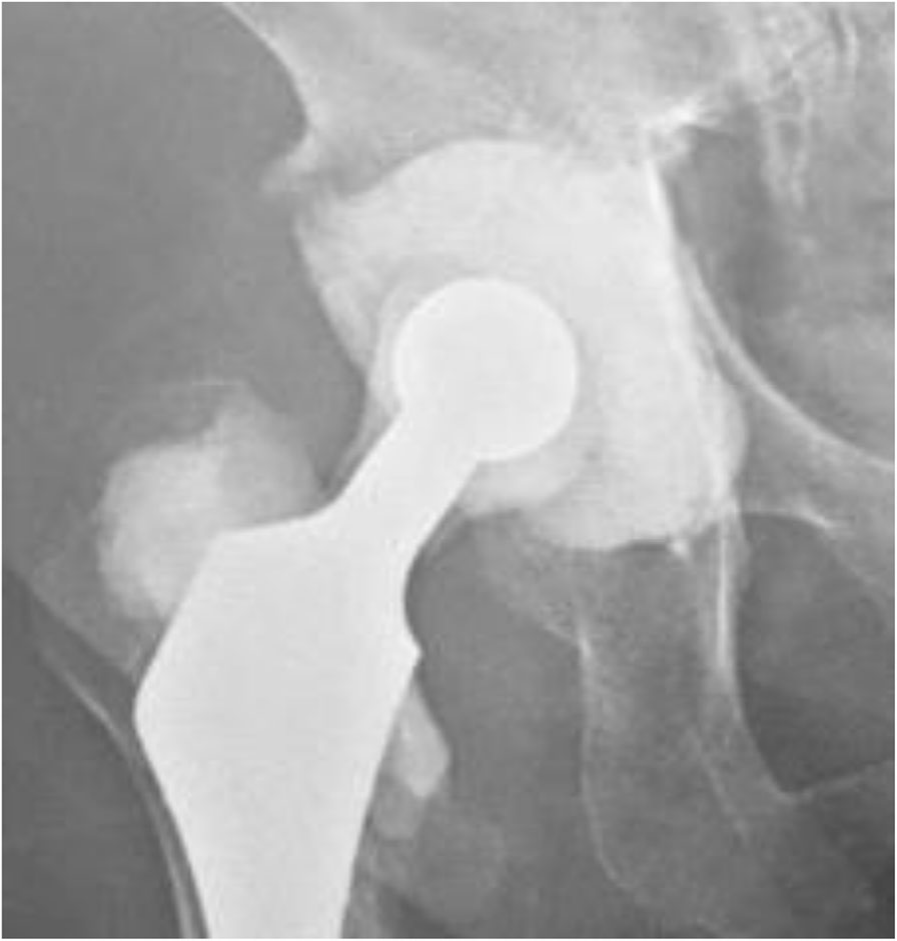
Recent trial to reduce the risk of dislocation; A larger (32 mm) diameter cement-on-cement articulating spacer was made for femoral and acetabular sides

Our literature search revealed five studies that reported the use of two-stage treatment with retention of well-fixed cementless stems [11–15], as in the present study. The number of patients was 1–19, the follow-up period was 3.5–4.6 years, and the infection control rate was 88–100% in these five studies. We further reported our clinical result of 5 patients followed up for an average of 9.1 years with an infection control rate of 100% (Table 1). Our study indicated the possibility of this treatment option for a long-term infection control. Other studies involving retention of well-fixed stems used a spacer shaped like a large-diameter artificial femoral head to articulate in the acetabular defect incurred during cup removal. However, in this study, we created a unique socket-like spacer because of a smaller risk of central migration of the spacer or acetabular fracture. The disadvantage of using a socket-like spacer is the associated risk of dislocation of the metal head from the cement spacer. Although none of the 6 patients had dislocation during the surgical interval in this study, to reduce the risk of dislocation, we performed cement-on-cement articulation using a large-diameter femoral head and socket-like spacer made of cement in recent clinical cases (Fig. 4).

**Table 1:** Results of two-stage treatment with retention of well-fixed cementless stem

Authors	No. of patients	Mean F/U (years)	Infection control rate
Anagnostakos et al. [11]	12	4.6	11/12 (92%)
Ekpo et al. [12]	19	4	17/19 (89%)
Faroug et al. [13]	1	3.5	1/1 (100%)
Fukui et al. [14]	5	4.2	5/5 (100%)
Lee et al. [15]	17	4	15/17 (88%)
Otani et al.	5	9.1	5/5 (100%)

The limitations of this study are the small number of cases, variation in the initial surgery, BHA, THA, and revision THA, which might make the difficulty of infection control different, and that indications for the current treatment method have not been clearly established. El-Husseiny et al. performed single-stage treatment to partially retain implants in carefully selected patients [17]. Indications for this treatment included good health, absence of sinus tract, and known causative bacteria, however MRSA infection was not a contraindication. The patients in our study were in relatively good health but included a case of MRSA infection accompanied by a sinus tract and cases with negative cultures. Because the effectiveness and indications of the present method are currently unclear, extreme caution should be exercised when the indication for PJI treatment is considered. In addition, comprehensive investigation is needed to evaluate factors such as local and systemic conditions of the patient, implant fixation status, and type of bacteria. Further study is warranted to accumulate cases to clarify the proper indications for retention of implants in treatment of PJI.

## Conclusions

The findings of this study suggest that potentially, in PJI patients with well-fixed and well-functioning cementless stem, two-stage treatment comprising stem retention and articulating cement spacer decreases local and systemic burden on the patient while preserving joint function and eradicating infection for a long-term. Indications for this treatment strategy require extremely careful consideration of factors such as local and general conditions of the patient, implant fixation status, and type of bacteria.

## Data Availability

This study was carried out in the Hospital of the Jikei University School of Medicine (3–25-8 Nishi-Shinbashi Minato-ku, Tokyo, Japan). The datasets used and/or analyzed during the current study are available from the corresponding author on reasonable request.

## Abbreviations

ADL: Activities of daily living
ALAC: Antibiotic-loaded acrylic cement
BHA: Bipolar hip arthroplasty
JOA score: Japanese Orthopaedic Association scoring system
MRSA: Methicillin-resistant *Staphylococcus aureus*
PJI: Periprosthetic joint infection
ROM: Range of motion
THA: Total hip arthroplasty
VCM: Vancomycin
